# Efficacy and Safety of CART Cell Therapy in Aggressive B‐Cell Lymphomas Involving the Gastrointestinal Tract

**DOI:** 10.1002/cnr2.70083

**Published:** 2025-01-28

**Authors:** Lixia Ma, Yimeng Dou, Rui Liu, Teng Xu, Fan Yang, Peihao Zheng, Shaomei Feng, Yuelu Guo, Hui Shi, Fei Xue, Biping Deng, Xiaoyan Ke, Kai Hu

**Affiliations:** ^1^ Department of Adult Lymphoma Beijing Boren Hospital Beijing China; ^2^ Cytology Laboratory Beijing Boren Hospital Beijing China

**Keywords:** blood malignancy, cancer management, cancer medicine, gastrointestinal bleeding, lymphoma

## Abstract

**Objective:**

Currently, chimeric antigen receptor T‐cell (CART) therapy represents a highly effective approach for relapsed/refractory B‐cell lymphomas. However, it also carries treatment‐related risks. Limited data are available on the risks associated with CART therapy in patients with gastrointestinal involvement in B‐cell lymphomas. Therefore, we conducted a retrospective cohort study to address this gap in knowledge.

**Methods:**

During the period from May 2019 to August 2022, a total of 26 patients recurrent/refractory with recurrent/refractory B‐cell lymphoma involving the gastrointestinal tract enrolled. Pathology confirmed CD19 antigen expression in tumor tissues. The disease status of patients who failed multiple lines of therapy was progressive disease (PD). Before CART cell infusion, patients received an FC regimen (fludarabine and cyclophosphamide) lymphodepletion. Quantitative PCR and flow cytometry were adopted for monitoring CART cell kinetics and function, with a focus on gastrointestinal AEs during treatment. The overall response rate (ORR) of the 26 patients was 61.5% (16/26), while the complete response rate (CR) was 23.1% (6/26). Their median follow‐up time was 22.49 months, while the medians of overall survival (OS) and progression‐free survival (PFS) were 10.88 and 5.47 months, respectively. The 1‐year OS and PFS rates were 45% and 42.3%, respectively. The prevalence of gastrointestinal complications was 21/26 (80.7%), including gastrointestinal hemorrhage in 11/26 (42.3%), emesis and diarrhea in 9/26 (34.6%), as well as intestinal obstruction in 2/26 (7.7%). A total of three patients (3/26, 11.5%) died of gastrointestinal hemorrhage. The gastrointestinal hemorrhage group exhibited markedly lower ORR and inferior OS compared to the non‐hemorrhage group.

**Conclusion:**

Generally, the CART cell therapy is valid in relapsed/refractory B‐cell lymphoma with gastrointestinal involvement, but gastrointestinal bleeding is a unique risk factor that requires special attention, particularly in patients with high gastrointestinal tumor burden, as it is associated with poor efficacy and survival.

## 1 Background

The gastrointestinal tract, which is the most usually affected site of extranodal non‐Hodgkin's lymphomas, represents 20%–40% of all such lymphomas [1, 2]. Among them, the incidence of diffuse large B‐cell lymphoma (DLBCL), the most frequent subtype, is approximately 47% [3]. Like other non‐Hodgkin's lymphomas, the R‐CHOP regimen is the extant regular first‐line therapy, whose 5‐year OR rate is about 50%–60% for B‐cell lymphoma involving the gastrointestinal tract [4]. Approximately 35% of patients progress to relapsed/refractory disease [5, 6, 7]. Due to the particular location of the lesions, gastrointestinal complications such as bleeding (9.8%–32.9%), perforation (9%), and intestinal obstruction (11.8%–17.4%) may occur during chemotherapy or due to disease progression and posttreatment tissue necrosis among both the therapeutically‐naive and refractory/recurrent populations [2, 8]. Like other relapsed/refractory B‐cell non‐Hodgkin's lymphomas, CART cell immunotherapy provides an important treatment option for individuals who had gastrointestinal involvement, but the incidence of gastrointestinal adverse events (AEs) during CART cell therapy and their impact on efficacy remains unclear, as patients with gastrointestinal involvement were not contained in most CAR‐T clinical trials.

Immune effector cell‐associated neurotoxicity (ICANS) and cytokine release syndrome (CRS) constitute the common AEs in CART cell immunotherapy for refractory/recurrent B‐cell non‐Hodgkin's lymphomas. Nevertheless, it may cause local inflammatory edema, exudation, and even bleeding in the lesion area due to the unique nature of CART cell therapy. Based on some relevant studies, this manifestation is called a localized CRS reaction after CART cell infusion in B‐cell non‐Hodgkin's lymphoma [9]. This may lead to serious gastrointestinal AEs, especially life‐threatening risks such as gastrointestinal bleeding and perforation, in patients with gastrointestinal involvement. Currently, there are no large‐scale studies describing the incidence of these AEs during cellular immunotherapy and their impact on efficacy. To clarify these issues, from 217 patients with refractory/recurrent B‐cell non‐Hodgkin's lymphomas who underwent CART cell immunotherapy in clinical trials, we extracted 26 patients showing gastrointestinal involvement and summarized their safety and efficacy, with a particular focus on gastrointestinal AEs.

## 2 Methods

### 2.1 Patient Inclusion

This study retrospectively analyzed 26 refractory/relapse B non‐Hodgkin's lymphomas (R/R B‐NHL) patients with gastrointestinal involvement from May 2019 to August 2022 at Beijing Boren Hospital, who participated in the clinical trial “Sequential Treatment of Relapsed/Refractory Adult Aggressive B‐cell Lymphoma with Different B‐cell Targeted CART Cells” (clinical research registration number: ChiCTR1900020980). Inclusion criteria: aged over 18 years; diagnosis of B‐cell non‐Hodgkin's lymphoma was confirmed in all patients according to the WHO 2016 criteria through endoscopic biopsy, puncture biopsy, or surgical removal of the lesion, and imaging examinations verified that the lesion involved the gastrointestinal tract; relapsed/refractory B‐cell non‐Hodgkin's lymphoma with at least two courses of first‐line and second‐line treatment (must use CD20‐targeted drugs and anthracycline drugs) without remission or relapse; pathological tissue immunohistochemistry or flow cytometry confirmed that tumor tissue expressed CD19 ≥ 90%. The approval of the current work was provided by the Ethics Committee of Beijing Boren Hospital. In addition, informed consent was acquired from every patient involved.

### 2.2 CART Cell Preparation and Pre‐Infusion Treatment

**FIGURE 1:**
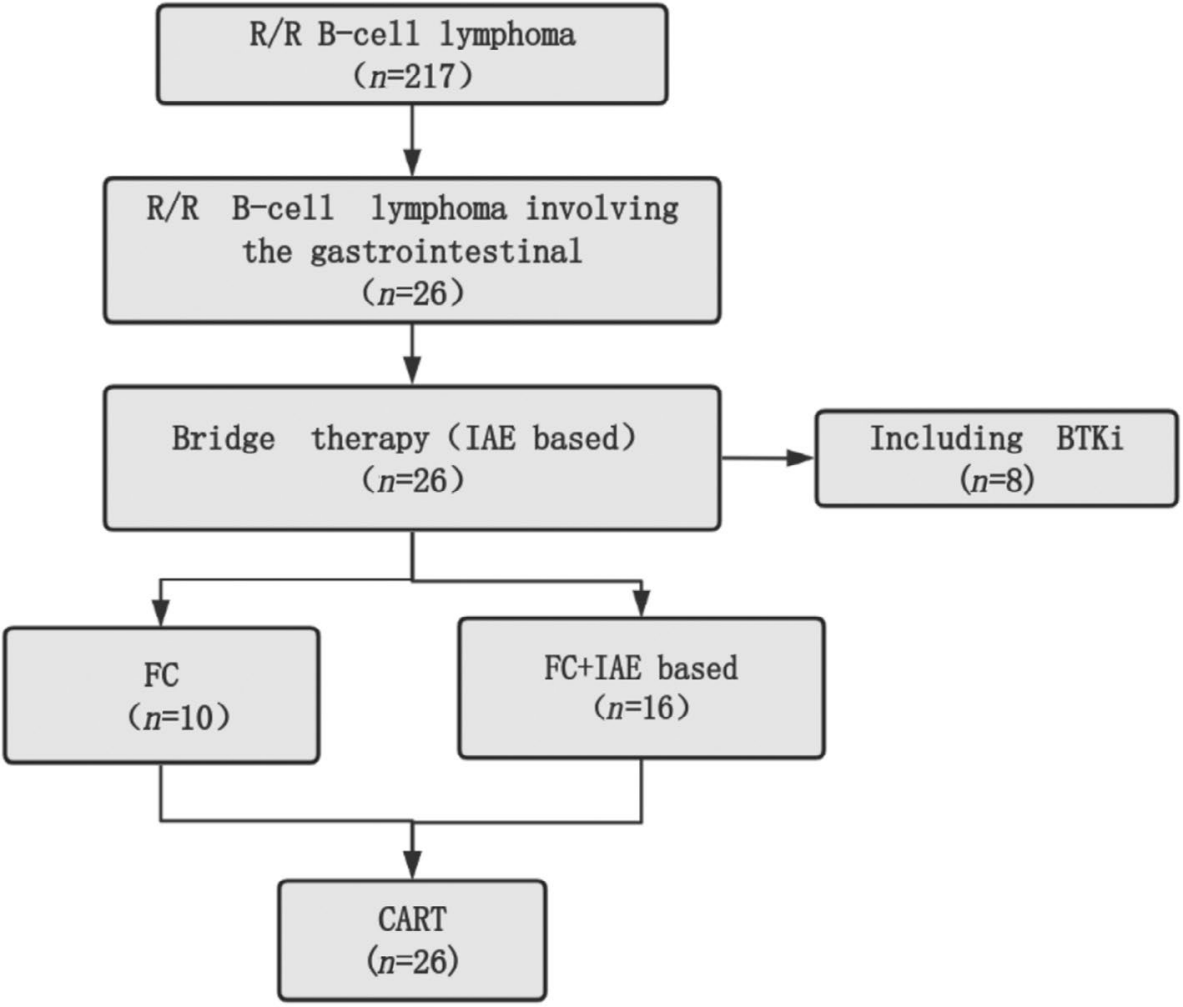
Flow diagram illustrating the treatment course in the study. BTKi, BTK inhibitor; FC, fludarabine and cyclophosphamide; IAE, ifosfamide, cytarabine, etoposide; R/R, recurrent/refractory.

After patient enrollment, single nucleated cells were isolated, and CD3+ immunomagnetic bead antigens were used to separate peripheral blood CD3+ T lymphocytes. The novel second‐generation CD19‐41BB‐lentiviral vector was constructed with the purpose of transfecting purified CD3+ T cells, constructing CD19‐targeted CART cells, as described in previous literature [10, 11, 12]. All patients underwent bridging chemotherapy prior to CART cell infusion, consisting IAE‐based regimen (ifosfamide, cytarabine, etoposide), aimed at controlling the disease and reducing tumor burden. Before CART cell infusion, all patients received fludarabine (30 mg/m^2^ from Day 5 to Day 3) and/or cyclophosphamide (250 mg/m^2^ from Day 5 to Day 3) as lymphodepleting preconditioning. Patients with high tumor burden (elevated LDH, presence of bulky, and high IPI scores) received additional IAE chemotherapy in conjunction with the FC regimen (Figure 1).

### 2.3 CART Kinetics and Cytokine Detection

Multicolor flow cytometry (BD FACSCalibur) was adopted for detecting the corresponding target CAR‐T cells, ELISA method was used for dynamically monitoring peripheral blood serum cytokines (IL‐6, IL‐10, TNF‐α, sCD25, IFN‐γ). Meanwhile, the chemiluminescence method was adopted for monitoring ferritin. Evaluations were performed on Days 0, 3, 7, 14, 21, and 28 following CART cell infusion and, subsequently, every month until 6 months, and every 3 months after 6 months of CART infusion until 24 months. Patients who do not achieve complete remission (CR) at the initial assessment may be considered for alternative salvage therapies.

### 2.4 Safety Observation

Therapy‐associated AEs, including ICANS (immune effector cells associated with neurologic toxicity) and CRS, were assessed for severity based on the ASTCT consensus grading system, and interventions were based on ASCO clinical guidelines and patient tolerance [13]. The severity of gastrointestinal AEs was evaluated in accordance with the National Cancer Institute CTCAE (version 5.0).

### 2.5 Gastrointestinal Adverse Reaction Management

Gastrointestinal bleeding is defined as positive fecal occult blood (visible red blood cells in routine stool tests), melena, and hematemesis, while excluding perianal issues such as hemorrhoids. All patients with gastrointestinal tract involvement were observed daily for gastrointestinal symptoms/abdominal examination and had a stool routine + occult blood test weekly. If gastrointestinal symptoms occurred, corresponding tests such as blood routine, coagulation, stool routine + occult blood, vomiting occult blood, and upright abdominal plain film were performed. For confirmed gastrointestinal bleeding, fasting, gastric tube placement (if necessary, for monitoring gastric fluid), ensuring enteral nutrition, acid‐suppressing proton pump inhibitors, somatostatin/octreotide, hemostasis, and coagulation factor supplementation were administered. For intestinal obstruction, fasting, ensuring enteral nutrition, and bowel movements were managed. In terms of patients without gastrointestinal symptoms, acid‐suppressing PPIs were managed to be used routinely.

### 2.6 Efficacy Evaluation

The efficacy was evaluated according to the NCCN guidelines and Lugano 2014 efficacy evaluation criteria [14, 15] with the best ORR/CR/PR, along with 1‐year rates of OS and PFS. Imaging examinations were accomplished on a monthly basis for the initial 6 months following CAR‐T cell infusion (including CT and ultrasound) and every 3 months thereafter with PET/CT until 24 months.

### 2.7 Statistical Analysis

Descriptive statistical methods were used to compare baseline demographic characteristics. Data analysis of binary variables was performed with the use of chi‐square and Fisher‐exact tests. With the use of *t*‐tests and rank sum tests, continuous variables were explored. SAS 9.4 software was used for statistical analyses. OS and PFS were analyzed by Cox proportional‐hazard regression model. The estimate of survival was calculated in accordance with the Kaplan–Meier method.

## 3 Results

### 3.1 Baseline Characteristics of the Patients

Twenty‐six patients with gastrointestinal tract involvement were enrolled and underwent CD19‐CART cell therapy between May 2019 and August 2022. The median age was 41 (19–69) years, and their male/female ratio was 1.8:1. DLBCL was diagnosed in 21 cases, while Burkitt lymphoma was diagnosed in five cases. The disease status was progressive, and at enrollment, all the patients were in stage III/IV. In addition, the median quantity of prior treatments was seven (2–27) cycles, encompassing autologous hematopoietic stem cell transplantation (auto‐HSCT) and radiotherapy each in 3/26 (11.5%). The median International Prognostic Index (IPI) score ≥ 3 accounted for 17/26 (65.4%). ECOG scores ≥ 3 accounted for 8/26 (30.8%). Gastrointestinal involvement features included: stomach (5/26, 19.2%), small intestine (2/26, 7.7%), colon (9/26, 35.0%), and multiple sites (10/26, 38.5%). 57.7% (15/26) patients had large masses (> 7.5 cm), 19.2% (5/26) patients had bone marrow involvement, 10.0% (3/26) patients were double/triple‐hit, and 61.5% (16/26) patients had TP53 mutations. Table 1 shows patient baseline characteristics.

**TABLE 1:** Demographic and clinical characteristics of the patients at baseline.

Characteristic	Number of cases, *n* (%)
Median age (range)—year	41 (19–69)
≧ 60	4 (15.4)
< 60	22 (84.6)
Sex (male:female)	1.8:1
Disease type	
DLBCL	21 (80.8)
Burkitt	5 (19.2)
COO classification in DLBCL	
Non‐GCB	14 (66.7)
GCB	7 (33.3)
IPI score	
< 3	9 (34.6)
≧ 3	17 (65.4)
ECOG performance‐status score	
< 3	18 (69.2)
≧ 3	8 (30.8)
Disease stage	
III/IV	26 (100%)
LDH	
Normal	9 (34.6)
High	17 (65.4)
Extranodal lesions (≧ 2)	23 (88.5)
Bulky disease (> 7.5 cm)	15 (57.7)
Bone marrow involvement	5 (19.2)
TP53 mutation	16 (61.5)
DH/TH	3 (11.5)
Prior treatment	
Autologous SCT with high‐dose CT	3 (11.5)
Chemotherapy cycles (median)	7 (2–27)
Radiotherapy	3 (11.5)
Involvement of the digestive tract	
Colon	9 (34.6)
Intestines	2 (7.7)
Stomach	5 (19.2)
Multi should read multiple sites	10 (38.5)
FC + IEA based	
Yes	16 (61.5)
No	10 (38.5)

Abbreviations: DH/TH, double hit/triple hit; DLBCL, diffuse large B‐cell lymphoma; ECOG, Eastern Cooperative Oncology Group; FC + IEA, fludarabine and cyclophosphamide + ifosfamide, cytarabine, etoposide; GCB, germinal center B cells; IPI, the International Prognostic Index; LDH, lactate dehydrogenase; non‐GCB, non‐germinal center B cells.

### 3.2 CART Cell Expansion and Kinetics

The median actual CART cell infusion value was 1.5 × 10^6^/kg (0.0711 × 10^6^/kg–4.46 × 10^6^/kg), with a median CART expansion peak number of 51/μL (0‐2890/μL). The median time to reach the CART peak was D11, and the median duration of CART in the body was 2 months. CART cell infusion dose and expansion peak made no significant effect on ORR, OS, or PFS (Table 2).

**TABLE 2:** Univariate analysis of outcomes (ORR, OS, and PFS).

	Optimal ORR			OS			PFS		
	HR	95% CI	*p*	HR	95% CI	*p*	HR	95% CI	*p*
Median age ≧ 60 years	6.4267	0.5634–73.5294	0.2642	2.132	0.583–7.813	0.2521	2.179	0.612–7.752	0.2292
Sex (male)	0.682	0.131–3.546	0.6924	0.588	0.203–1.703	0.3279	0.715	0.253–2.016	0.5255
Disease type (Burkitt)	3	0.4035–22.3025	0.3402	1.43	0.397–5.143	0.5841	1.195	0.336–4.255	0.7833
COO_classification (non‐GCB)	1.3889	0.1935–9.9675	1	1.068	0.312–3.659	0.9163	0.938	0.282–3.119	0.9164
IPI score ≧ 3	9.0009	0.915–88.496	0.0873	2.899	0.803–10.417	0.1041	2.0	0.633–6.329	0.2378
ECOG score ≧ 3	10.5042	1.497–73.529	0.0256	2.825	0.958–8.333	0.0598	2.1053	0.737–6.024	0.1646
High LDH	3.111	0.495–19.531	0.3989	1.697	0.529–5.441	0.3735	1.746	0.55–5.538	0.3443
Extranodal lesions ≧ 2	2.077	0.185–23.31	1	1.208	0.265–5.405	0.805	1.433	0.322–6.369	0.6365
Bulky disease ≧ 7.5 cm	2.333	0.439–12.392	0.4279	1.419	0.474–4.251	0.5314	1.792	0.608–5.28	0.29
BM involvement	10	0.919–108.696	0.0549	2.798	0.836–9.362	0.095	2.079	0.652–6.632	0.2163
TP53 mutation	0.303	0.038–2.421	0.3254	0.731	0.193–2.755	0.6446	0.674	0.181–2.506	0.556
DH/TH	3.750	0.293–48.077	0.5385	2.018	0.445–9.152	0.3629	1.416	0.318–6.297	0.648
Frequency of chemotherapy	—	—	0.4037	1.038	0.935–1.151	0.4863	1.124	0.985–1.283	0.0821
Colon involvement	0.321	0.0512–2.019	0.3989	0.401	0.112–1.442	0.1618	0.585	0.186–1.84	0.3589
Intestines involvement	1.667	0.092–30.030	1	0.921	0.12–7.065	0.9372	0.913	0.12–6.965	0.9304
Stomach involvement	1.083	0.147–7.962	1	0.628	0.14–2.814	0.5434	0.523	0.118–2.322	0.3944
Multi should read multiple sites' involvement	2.200	0.431–11.223	0.425	3.018	1.038–8.775	0.0425	2.469	0.892–6.828	0.0817
FC + IEA based	4	0.640–25	0.2177	0.989	0.341–2.867	0.9835	0.913	0.329–2.532	0.8611
Gastrointestinal bleeding after CART cell therapy	17.331	2.360–126.582	0.004	3.609	1.228–10.605	0.0196	2.882	1.021–8.132	0.0455
CRS grade ≧ 3	1.857	0.293–11.751	0.6443	1.757	0.549–5.623	0.3423	1.406	0.446–4.431	0.5611
The actual CART cell infusion value	—	—	0.1449	0.821	0.48–1.403	0.4699	0.753	0.446–1.273	0.2897
CART expansion peak	—	—	0.4420	1	1–1.001	0.5572	1	0.999–1.001	0.7317
Gastrointestinal bleeding prior to CART	14.993	1.397–161.290	0.0184	4.088	1.327–12.592	0.0142	3.535	1.167–10.71	0.0256
Bridging the use of BTKi	10.504	1.497–73.529	0.0256	3.321	1.13–9.75	0.029	2.421	0.851–6.887	0.0975

Abbreviations: CART, chimeric antigen receptor T‐cell; DH/TH, double hit/triple hit; DLBCL, diffuse large B‐cell lymphoma; ECOG, Eastern Cooperative Oncology Group; FC + IEA, fludarabine and cyclophosphamide + ifosfamide, cytarabine, etoposide; GCB, germinal center B‐cell like; IPI, the International Prognostic Index; LDH, lactate dehydrogenase; non‐GCB, non‐germinal center B‐cell like; ORR, overall response rate; OS, overall survival; PFS, progression‐free survival.

### 3.3 Efficacy

**FIGURE 2:**
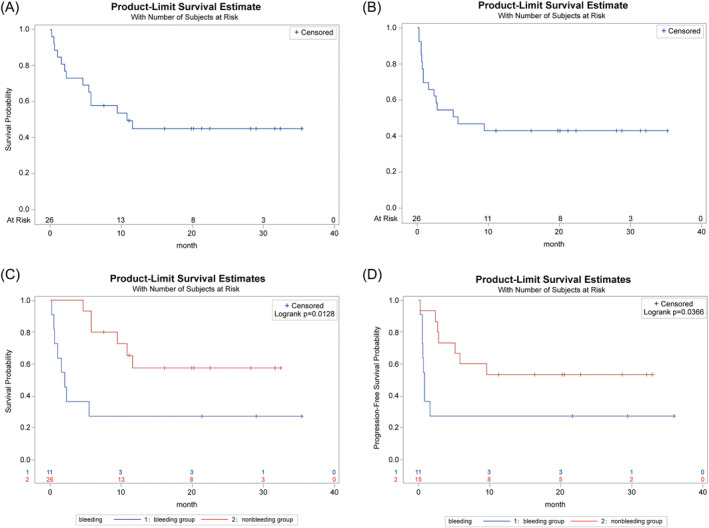
Survival curve. Total OS (A), total PFS (B); bleeding group, non‐bleeding group OS (C), PFS (D). PFS, progression‐free survival; OS, overall survival.

The optimal overall response rate (ORR) within 90 days was 61.5% (16/26) for the 26 patients, whereas the complete response rate (CR) was 23.1% (6/26). Their median follow‐up time was 22.49 (0.23–35.44) months, while the medians of overall survival (OS) and progression‐free survival (PFS) were 10.88 and 5.47 months, respectively (Figure 2). ECOG score ≥ 3 (*p* = 0.0256), gastrointestinal bleeding symptoms prior to CART (*p* = 0.0184), and bridging use of BTKi (*p* = 0.0256) were related to a lower ORR. Gastrointestinal involvement at multiple sites (*p* = 0.0425), gastrointestinal bleeding before CART (*p* = 0.0142), and bridging use of BTKi (*p* = 0.029) were related to shorter OS. Gastrointestinal bleeding symptoms prior to CART (*p* = 0.0455) and gastrointestinal bleeding symptoms after CART were both associated with shorter PFS (*p* = 0.0256). Other baseline factors made no statistically significant effect on the best ORR, OS, or PFS (Table 2).

**FIGURE 3:**
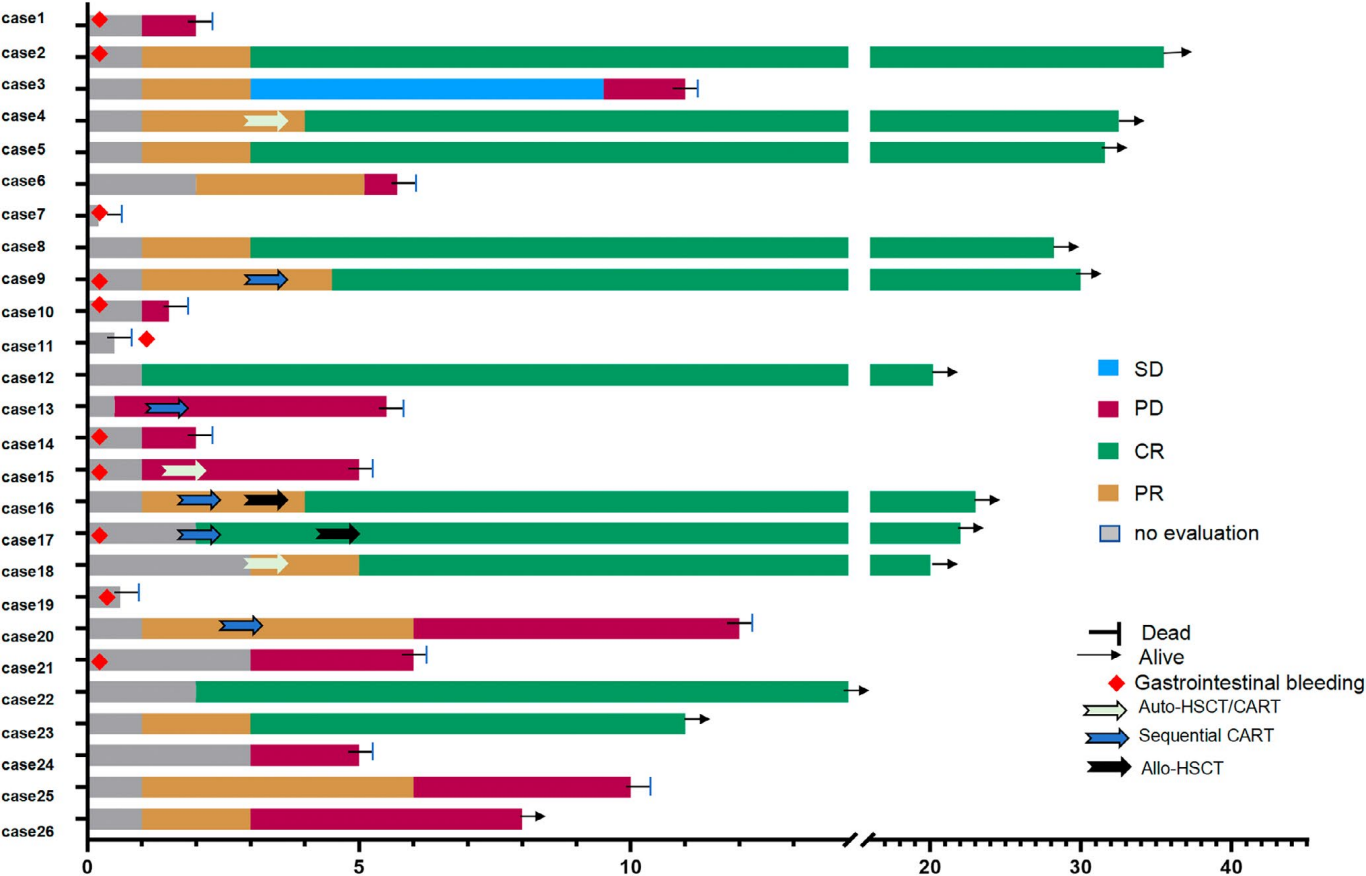
Swimmer plot illustrating the clinical response and follow‐up of patients treated with the proposed regimen. AlloHCT, allogeneic hematopoietic cell transplantation; Auto‐HSCT, autologous chondrocyte transplantation; CART, chimeric antigen receptor T‐cell; CR, complete response; PD, progressive disease; SD, stable disease.

Until the cut‐off time, there were still 11 patients alive and in CR, one patient was surviving with disease, and 14 patients had passed away, with three dying from post‐CART bleeding and 11 from disease progression. Patients who did not achieve CR at the initial assessment subsequently received salvage therapy. Three patients (Case 4, Case 15, Case 18) who did not achieve CR at the initial assessment after CART cell therapy underwent auto‐HSCT followed by CAR‐T cell immunotherapy. As of the last follow‐up, two patients remained in remission while one patient died due to disease progression. Additionally, five patients who did not achieve CR at the initial assessment after CART cell therapy received CAR‐T cell immunotherapy targeting other antigens as salvage therapy. Among them, two patients (Case 16, Case 17) underwent allogeneic hematopoietic stem cell transplantation. As of the last follow‐up, three patients remained in remission while two patients died due to disease progression (Figure 3).

### 3.4 Safety

Gastrointestinal complications occurred in 21/26 (80.8%) patients, containing gastrointestinal bleeding in 11/26 (42.3%), intestinal obstruction in 2/26 (7.7%), and vomiting and diarrhea in 9/26 (34.6%). After CART infusion, the median time to gastrointestinal bleeding was 4 days (range 1–33 days). The median duration of bleeding was 11 days (range 4–30 days). Among the 11 cases of gastrointestinal bleeding, five (5/11, 45.5%) had positive fecal occult blood, and six (6/11, 54.5%) had overt gastrointestinal bleeding (including hematochezia, hematemesis, and melena). Three patients died due to gastrointestinal bleeding: One (Case 7) was a 67‐year‐old male with DLBCL involving the stomach and colon who had positive fecal occult blood before infusion, no other gastrointestinal symptoms, and sudden hematemesis of approximately 700 mL on D7 after infusion, followed by continuous bleeding, hypotension, and death despite resuscitation efforts. One patient (Case 11) was a 38‐year‐old male with BL involving extensive intestines who developed dark red bloody stools and hypotension on D5, received aggressive medical hemostasis and supportive treatment, but continued to have bloody stools. On D14, mesenteric angiography was performed but no culprit vessel was found for surgical intervention, and the patient died on D16. One patient (Case 19) was a 41‐year‐old female with DLBCL involving the small intestine and colon who experienced melena and fresh blood‐like stools 11 days after tumor reduction chemotherapy, with an estimated volume of 3000 mL, hypotension, and aggressive medical hemostasis and supportive treatment. Bleeding was controlled, and after fecal occult blood turned negative, CART cells were infused on a compassionate basis. On D12, the patient experienced watery diarrhea with positive fecal occult blood, which improved after conservative medical treatment. On D18, the patient developed melena with an estimated volume of 1500 mL, accompanied by abdominal pain and distension, hypotension, and died despite resuscitation efforts. Two patients experienced diarrhea, which was confirmed by stool culture due to *Rhodotorula mucilaginosa* and *Pseudomonas aeruginosa* infections.

In four out of six cases of overt gastrointestinal bleeding, recombinant human coagulation factor VIIa was administered, with a median application time of 11 days (range: 1–33 days) and a median total dose of 9 mg (range: 4–33 mg). The bleeding ceased in two patients after administration, while the response was poor in the other two patients. Six patients had gastrointestinal bleeding before CART cell therapy (two cases of overt bleeding and four cases of positive fecal occult blood); three cases of fecal occult blood turned negative before CART cell infusion, while the remaining three cases remained positive. In four patients, fecal occult blood turned negative after CART cell therapy.

Gastrointestinal hemorrhage following CART cell therapy was associated significantly with inferior PFS (*p* = 0.0455), OS (*p* = 0.0196), as well as ORR (*p* = 0.004) (Table 2). The gastrointestinal bleeding group displayed markedly lower ORR compared to the non‐bleeding group (3/11, 27.3% vs. 13/15, 86.7%) (*p* = 0.0040). The gastrointestinal bleeding group had lower PFS in relative to the non‐bleeding group (*p* = 0.0366), with a median PFS of 5.12 months in the bleeding group. Contrastively, the median follow‐up duration was not reached in the non‐bleeding group. The gastrointestinal bleeding group exhibited markedly lower OS compared to the non‐bleeding group (*p* = 0.0128), where the median OS was 10.88 months. Contrastively, the non‐bleeding group did not reach the median follow‐up time (Figure 2). Regarding median peak levels and infusion dose of CAR‐T cells in vivo, there existed no obvious differences between the two groups (Table 4).

**FIGURE 4:**
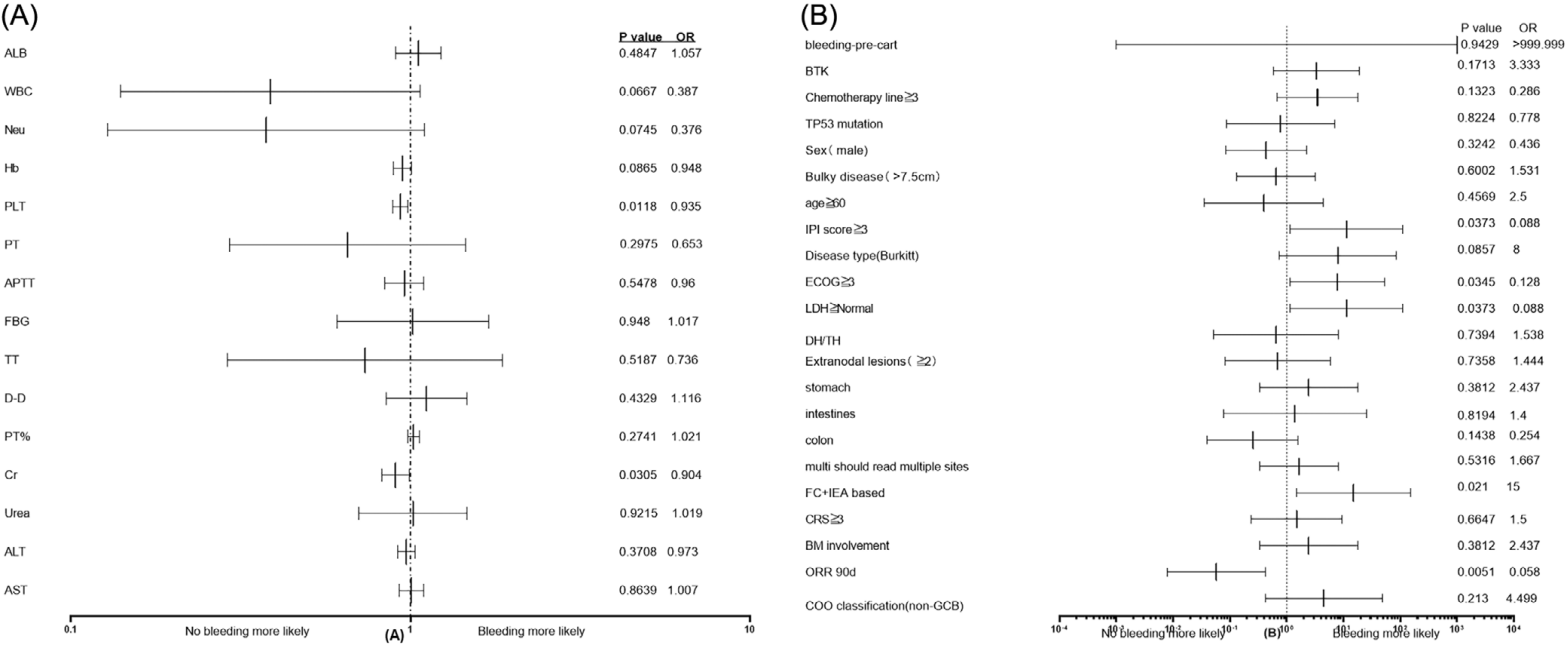
Statistical analysis of bleeding risk factors. Laboratory (A) and baseline (B) pairs univariate analysis of gastrointestinal bleeding.

Due to the significant impact of gastrointestinal bleeding on the efficacy and survival after CART cell immunotherapy, an analysis of the bleeding risk factors was performed. Patients with a history of gastrointestinal bleeding symptoms before CART therapy are more likely to experience gastrointestinal bleeding after CART therapy., compared with the patients without gastrointestinal bleeding history (*p* = 0.002). The patients who received FC + IEA‐based bridging therapy are more prone to gastrointestinal bleeding after CART treatment, comparing to FC group (10/11, 90.9% vs. 6/15, 40%) (*p* = 0.0143). Patients with an ECOG score of ≥ 3 were markedly higher in proportion in the bleeding group than in the non‐bleeding group (6/11, 54.5% vs. 2/15, 13.3%) (*p* = 0.0384). Besides, the bleeding group contained prominently more patients with an IPI score of ≥ 3 compared to the non‐bleeding group (10/11, 90.9% vs. 8/15, 53.3%) (*p* = 0.0362). Patients with high LDH levels were significantly higher in proportion in the hemorrhage group than in the non‐hemorrhage group (10/11, 90.9% vs. 7/15, 46.7%) (*p* = 0.0362) (Table 3). Univariate analysis revealed that decreased platelet count, lower serum creatinine levels within the normal range, IPI ≥ 3, ECOG ≥ 3, elevated LHD, and FC + IAE‐based lymphodepletion were high‐risk factors for gastrointestinal bleeding after CAR‐T therapy (Figure 4).

**TABLE 3:** Demographic and clinical characteristics of the bleeding group and non‐bleeding group at baseline.

Characteristic	Bleeding group (*n* = 11)	Non‐bleeding (*n* = 15)	*p*
Median age (range)—year	40 (29–67)	45 (19–69)	0.6137
≧ 60			
< 60			
Sex (male:female)	1.2:1	2.75:1	0.4185
Disease type			
DLBCL	7 (33.3)	14 (66.7)	0.1279
Burkitt	4 (80.0)	1 (20.0)	
COO classification in DLBCL			
Non‐GCB	6 (85.7)	8 (57.1)	0.337
GCB	1 (14.3)	6 (42.9)	
IPI score			
≧ 3	10 (58.8)	7 (41.2)	0.0362
< 3	1 (11.1)	8 (88.9)	
ECOG performance‐status score			
≧ 3	6 (75.0)	2 (25.0)	0.0384
< 3	5 (27.8)	13 (72.2)	
Disease stage			
III/IV	11 (42.3)	15 (57.7)	
LDH			
≦ abnormal	10 (58.8)	7 (41.3)	0.0362
> abnormal	1 (11.1)	8 (88.9)	
Extranodal lesions (≧ 2)	10 (43.5)	13 (56.5)	1.0000
Bulky disease (> 7.5 cm)	7 (46.7)	8 (53.3)	0.7007
Bone marrow involvement	3 (60.0)	2 (40.0)	0.6196
TP53 mutation	7 (43.8)	9 (56.3)	1.0000
DH/TH	1 (33.3)	2 (66.7)	1.0000
Prior treatment			
Autotransplantation	1 (33.3)	2 (66.7)	
Chemotherapy cycles (median)	7 (2–17)	8 (2–27)	0.9168
Involvement of the digestive tract			1.0000
Colon	2 (22.2)	7 (77.8)	0.2167
Intestines	1 (50.0)	1 (50.0)	1.0000
Stomach	3 (60.0)	2 (40.0)	0.6196
Multi	5 (50.0)	5 (50.0)	0.6891
Gastrointestinal bleeding prior to CART			
Yes	6 (54.5)	0	0.002
No	5 (45.5)	15 (100)	
FC + IEA based			
Yes	10 (62.5)	6 (37.5)	0.0143
No	1 (10.0)	9 (90.0)	
Bridging the use of BTKi			
Yes	5 (45.5)	3 (20)	
No	6 (54.5)	12 (80)	

Abbreviations: CART, chimeric antigen receptor T‐cell; DH/TH, double hit/triple hit; DLBCL, diffuse large B‐cell lymphoma; ECOG, Eastern Cooperative Oncology Group; FC + IEA, fludarabine and cyclophosphamide + ifosfamide, cytarabine, etoposide; GCB, germinal center B cells; IPI, the International Prognostic Index; LDH, lactate dehydrogenase; non‐GCB, non‐germinal center B cells.

**TABLE 4:** The median actual CAR‐T cell infusion value and median CART expansion peak.

	All (*n* = 26)	Bleeding group (*n* = 11)	Non‐bleeding (*n* = 15)	*p*
Median actual CART cell infusion value, 106/kg	1.5 (0.0711–4.46)	1.73 (0.27–2.9)	1.39 (0.0711–4.46)	0.7752
Median CART expansion peak, μL	51 (0–2890)	43 (0–369)	51 (0–2890)	0.8550

Abbreviation: CART, chimeric antigen receptor T‐cell.

**TABLE 5:** Incidence of CRS and ICANS.

	Number of cases, *N* (%)	Bleeding group (*n* = 11), *N* (%)	Non‐bleeding (*n* = 15), *N* (%)	*p*
CRS 1–5	24 (92.3)	11 (45.8)	13 (54.2)	0.4923
CRS ≧ 3	6 (25)	5 (83.3)	1 (16.7)	1.0000
ICANA 1–5	2	2	0	—
ICANA ≧ 3	2	2	0	—

Abbreviations: CRS, cytokine release syndrome; ICANS, immune effector cell‐associated neurotoxicity.

The prevalence of any grade CRS was 92.3% (24/26), of which severe CRS (≥ Grade 3) accounted for 25% (6/24). The morbidity of ≥ Grade 3 CRS had no statistical significance between the gastrointestinal bleeding and non‐bleeding groups (*p* = 1.0000). No difference between the two groups was noted also in the morbidity of ICANS (Table 5).

## 4 Discussion

Gastrointestinal involvement in relapsed/refractory DLBCL is a commonly seen clinical subtype. Previous literature has reported that gastrointestinal non‐Hodgkin lymphoma (NHL) is more common in men aged 50–60 years, primarily occurring as a secondary malignancy, accounting for 10%–15% of all NHLs. The majority of these are B‐cell NHLs, with DLBCL being the most common subtype. The most frequently affected site is the stomach (60%–75%), followed by the small intestine (20%–30%), colon (10%–20%), and multiple sites (6.5%) [16, 17, 18, 19]. The patient cohort in our report had advanced‐stage gastrointestinal involvement (all stage III/IV), with the majority being non‐GCB DLBCL (66.7%), an IPI > 3 (65.4%), and TP53 mutations (61.5%). Gastrointestinal multisite involvement and colon involvement were most common. The pathological type in our cohort is consistent with the literature, and as a group of relapsed/refractory patients, high tumor burden and high TP53 mutations may be characteristic features of their relapse and refractoriness.

Relapsed/refractory B‐cell NHL CART cell immunotherapy remains a potential alternative treatment option. Traditional chemotherapy treatments include chemotherapy, autologous hematopoietic stem cell transplantation, allogeneic hematopoietic stem cell transplantation, as well as radiotherapy. Previous literature has reported that the ORR for second‐line chemotherapy in relapsed/refractory B‐cell NHL ranges from 26% to 63.5%, with a CR rate of 7%–27% [20, 21, 22, 23, 24]. Chemotherapy‐sensitive patients have an ORR of 44%–84% and a CR rate of approximately 27% following autologous transplantation [25, 26, 27, 28]. Among the 19% of patients who relapse after autologous transplantation, the ORR for allogeneic hematopoietic stem cell transplantation is 49%, with a CR rate of around 5.5%; 51% of these patients experience rapid disease progression [24, 25]. Most patients are not eligible for autologous hematopoietic stem cell transplantation, and the ORR for those who are ineligible is 12%–23% [25, 26, 27, 28]. CAR‐T cell immunotherapy is considered as a novel treatment option for relapsed/refractory B‐cell NHL, with ORR ranging from 52% to 82% and CR rates of 40%–57% in international studies [29, 30, 31]. In our center, the ORR for CART cell immunotherapy in relapsed/refractory B‐cell NHL is 58.0%, with a CR rate of 42.8% [32]. The effectiveness of CART cell immunotherapy for gastrointestinal lymphoma has also been confirmed by research. A study by Zeng et al., which included 14 patients with gastrointestinal B‐cell lymphoma, showed an ORR of 71.4% and a CR rate of 50% for CAR‐T‐cell immunotherapy in relapsed/refractory gastrointestinal B‐cell NHL [33]. Albert et al. presented an ORR of 63% and a CR rate of 42% for CD19‐targeted CART therapy in 24 patients with gastrointestinal lymphoma [34]. In our cohort of 26 patients, the best overall ORR and CR rates for CART cell therapy were 61.5% and 23.1%, respectively. It is clear that the efficacy of CART cell immunotherapy in this group of 26 patients is significantly better than that of traditional chemotherapy. All patients in our cohort were stage III/IV, had multisite gastrointestinal involvement, large tumor masses, extranodal involvement, and were refractory to multiple lines of therapy. The best ORR of 61.5% for CART cell immunotherapy in our cohort is comparable to the literature, and the CR rate of 23.1% suggests that CART cell therapy is overall effective for patients with gastrointestinal involvement.

Due to the unique anatomical location of gastrointestinal lymphomas, there may be specific safety concerns correlated with CART cell therapy. In a clinical trial of continuous infusion of anti‐CD19 and anti‐CD22 CART cells, Zeng et al. reported gastrointestinal AEs in 14 patients with gastrointestinal B‐cell lymphoma, with the most common being diarrhea (4/14), bleeding (2/14), and no perforations [33]. Albert et al. reported gastrointestinal AEs in CD19‐targeted CART therapy for gastrointestinal lymphoma, with the most common being gastrointestinal perforation (3/24) [34]. These two clinical studies on gastrointestinal B‐cell lymphoma CART cell immunotherapy did not report the incidence of gastrointestinal bleeding and its impact on efficacy. In our cohort of 26 patients, the incidence of gastrointestinal complications was 80.7%, with gastrointestinal bleeding in 42.3% (11/26), intestinal obstruction in 7.7%, and vomiting and diarrhea in 30.8%; three out of 26 patients died due to gastrointestinal bleeding. Gastrointestinal bleeding significantly affected ORR, OS, and PFS. The main reasons for the high incidence of gastrointestinal bleeding after CART cell therapy in this cohort were high tumor burden, severe local CRS [9], increased detection of fecal occult blood leading to a higher rate of positive fecal occult blood tests, and the use of steroids and non‐steroidal anti‐inflammatory drugs during the management of CRS and ICANS leading to an increased risk of gastrointestinal bleeding.

Factors related to high tumor burden and thrombocytopenia during treatment may be associated with gastrointestinal bleeding. In our report on single‐factor analysis of gastrointestinal bleeding in patients with gastrointestinal involvement undergoing CART cell immunotherapy, we found that decreased platelet count, lower creatinine levels within the normal range, IPI ≧ 3, ECOG ≧ 3, elevated LDH, and FC + IAE‐based lymphodepletion were factors contributing to gastrointestinal bleeding after CART treatment. High IPI scores, elevated LDH levels, and high ECOG scores have been reported to negatively impact prognosis, indicating that patients with high tumor burden and poor general condition need to be particularly vigilant about gastrointestinal bleeding. Decreased platelet count is an independent risk factor for all hemorrhagic diseases, and both chemotherapy before CART cell infusion and bone marrow suppression after infusion can lead to a decrease in platelet count. For patients with low pre‐infusion platelet levels, transfusion of platelets and the use of drugs promoting platelet survival can correct thrombocytopenia and reduce the risk of bleeding. Lower creatinine levels within the normal range may be related to poor nutritional status and are often associated with high gastrointestinal tumor burden. The proportion of patients with gastrointestinal bleeding significantly increased after FC + IAE‐based lymphodepletion, mainly because patients receiving tumor reduction therapy often have large tumors and high tumor burden; secondly, bone marrow suppression before infusion on the basis of the FC regimen results in even lower platelet levels, indicating that conventional tumor reduction chemotherapy cannot reduce the risk of bleeding. There is a risk of bleeding with BTKi therapy. Bleeding of any grade has an overall incidence of 40% [35]. Only four patients (4/11) in the gastrointestinal bleeding group received tumor reduction chemotherapy plus BTKi inhibitor. In our cohort of patients, most gastrointestinal bleeding was controlled with preventive acid suppression (proton pump inhibitors), hemostatic agents (phenolsulfonethamine, carbesulphane), supplementation of coagulation factors (fibrinogen, prothrombin complex, recombinant human coagulation factor VIIa, etc.) and symptomatic support (transfusion of blood components and nutrition), but three patients still died of bleeding. In addition to conservative internal medicine treatments for preventing and treating bleeding, it remains to be seen whether interventional procedures, radiotherapy, or surgery can be incorporated for high‐risk patients to improve the safety and effectiveness of treatment, but there are currently no related studies.

Gastrointestinal bleeding has adverse correlations with both treatment efficacy and patient prognosis, in addition to ECOG scores and tumor burden also being adverse prognostic factors. A history of gastrointestinal bleeding symptoms before CART therapy and bridging therapy including BTKi has an impact on both ORR and OS. A history of gastrointestinal bleeding symptoms affects PFS, while an ECOG score > 3 affects ORR, and multiple gastrointestinal site involvement affects OS. These findings conform to previous reports on the impact of tumor burden and patient status on treatment efficacy and survival. It also suggests that patients with gastrointestinal bleeding prior to CART treatment have a significantly increased risk of post‐CART bleeding, which in turn affects treatment efficacy and long‐term survival. Previous studies have shown that common adverse reactions to CART cell immunotherapy include CRS and ICANS, with incidence rates of 42%–93% and 20%–64%, respectively. Grade ≥ 3 CRS reactions occur in 2%–22% of cases, while Grade ≥ 3 ICANS reactions occur in 10%–28% [29, 30, 31]. Our study shows that the incidence rates of CRS and ICANS and their rates of Grade ≥ 3 reactions are generally consistent with previous reports.

However, the current work still has the following limitations: it is a single‐center retrospective study that has a comparatively small sample size and a short follow‐up period. In our future work, we need to accumulate more cases and conduct long‐term follow‐ups for verification.

## 5 Conclusion

Our study shows that CAR‐T cell therapy is generally valid for relapsed/refractory B‐NHL patients with gastrointestinal involvement, but gastrointestinal bleeding significantly reduces its efficacy and survival. There is a need for heightened vigilance for gastrointestinal bleeding during CAR‐T cell therapy and for the prevention and therapy of gastrointestinal bleeding in patients with high‐risk factors.

## Author Contributions

**Lixia Ma:** conceptualization, writing – original draft. **Yimeng Dou:** methodology. **Rui Liu:** methodology, investigation. **Teng Xu:** investigation, methodology. **Fan Yang:** investigation, software. **Peihao Zheng:** investigation, data curation. **Shaomei Feng:** investigation, methodology. **Yuelu Guo:** conceptualization, methodology. **Hui Shi:** conceptualization, writing – review and editing. **Fei Xue:** conceptualization, writing – review and editing. **Biping Deng:** conceptualization, software. **Xiaoyan Ke:** writing – review and editing, conceptualization. **Kai Hu:** writing – review and editing, conceptualization, writing – original draft.

## Conflicts of Interest

The authors declare no conflicts of interest.

## Data Availability

The data that support the findings of this study are available in the main manuscript.
